# *Rickettsia parkeri* Rickettsiosis, Arizona, USA

**DOI:** 10.3201/eid2205.151824

**Published:** 2016-05

**Authors:** Kristen L. Herrick, Sandra A. Pena, Hayley D. Yaglom, Brent J. Layton, Amanda Moors, Amanda D. Loftis, Marah E. Condit, Joseph Singleton, Cecilia Y. Kato, Amy M. Denison, Dianna Ng, James W. Mertins, Christopher D. Paddock

**Affiliations:** Arizona Department of Health Services, Phoenix, Arizona, USA (K.L. Herrick, H.D. Yaglom);; Gila County Division of Health and Emergency Management, Globe, Arizona, USA (S.A. Pena);; Pinal Mountain Internal Medicine, Globe (B.J. Layton);; Moors Wildlife Management Services, Globe (A. Moors);; Midwestern University, Glendale, Arizona, USA (A.D. Loftis);; Centers for Disease Control and Prevention, Atlanta, Georgia, USA (M.E. Condit, J. Singleton, C.Y. Kato, A.M. Denison, D. Ng, C.D. Paddock);; National Veterinary Services Laboratories, Ames, Iowa, USA (J.W. Mertins)

**Keywords:** rickettsial, rickettsiosis, Rickettsia parkeri, Amblyomma triste, Amblyomma maculatum, eschar, vector-borne infections, ticks, Arizona, bacterial infection, bacteria

## Abstract

In the United States, all previously reported cases of *Rickettsia parkeri* rickettsiosis have been linked to transmission by the Gulf Coast tick (*Amblyomma maculatum*). Here we describe 1 confirmed and 1 probable case of *R. parkeri* rickettsiosis acquired in a mountainous region of southern Arizona, well beyond the recognized geographic range of *A. maculatum* ticks. The likely vector for these 2 infections was identified as the *Amblyomma triste* tick, a Neotropical species only recently recognized in the United States. Identification of *R. parkeri rickettsiosis* in southern Arizona demonstrates a need for local ecologic and epidemiologic assessments to better understand geographic distribution and define public health risk. Education and outreach aimed at persons recreating or working in this region of southern Arizona would improve awareness and promote prevention of tickborne rickettsioses.

## Abstract 2

The likely vector was *Amblyomma triste*, a Neotropical tick species only recently recognized in the United States.

*Rickettsia parkeri*, a tickborne bacterium that causes a febrile, eschar-associated illness throughout many countries of the Western Hemisphere, is transmitted by *Amblyomma* ticks. In the United States, ≈40 cases of *R. parkeri* rickettsiosis have been reported since its recognition in 2004 (*1*). The Gulf Coast tick (*Amblyomma maculatum*) is the principal vector of *R. parkeri* in the United States (*2*), and all previously documented US infections arose within the known geographic range of these ticks (*1*). Confirmed cases of *R. parkeri* rickettsiosis also have been reported from Uruguay and Argentina, where *A. triste* and *A.* *tigrinum* ticks serve as the principal vector species (*3*–*6*). Recent reviews of tick collection records and archived specimens documented and identified the presence of ticks very closely related to *A. triste* in several regions of the southwestern United States and adjacent regions of Mexico since at least 1942 (*7*,*8*). Here we report 1 confirmed and 1 probable case of *R. parkeri* rickettsiosis, each acquired in southern Arizona after bites from *A. triste* ticks.

## Case Histories

### Patient 1

**Figure 1:**
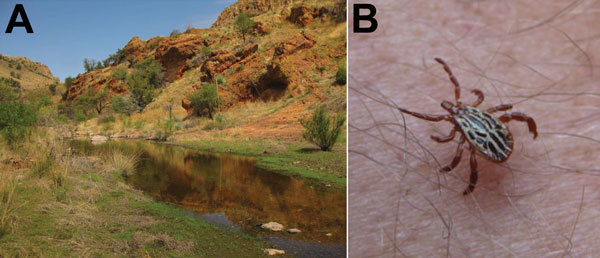
A) Typical habitat in the Pajarito Mountains in Santa Cruz County, Arizona, USA, near the location where patient 1 sustained a bite from a tick that resulted in *Rickettsia parkeri* rickettsiosis in July 2014. B) Male tick identical to the tick that bit patient 1. The distinctive white ornamentation on the scutum and disjunct geographic origin strongly support its presumptive identification as *Amblyomma triste*.

**Figure 2:**
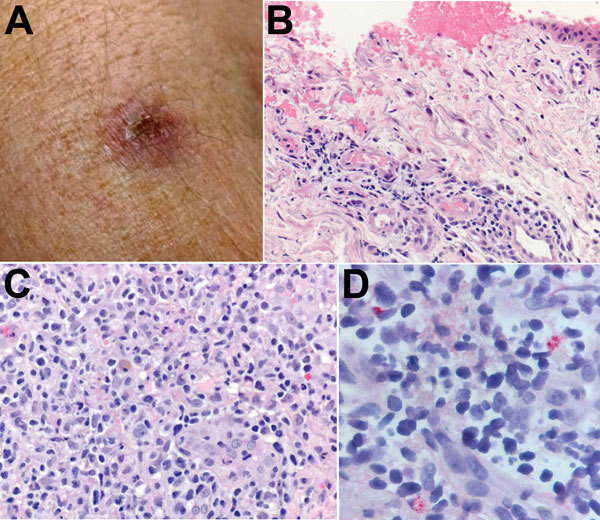
A) Eschar on the right arm of patient 1 at the site of tick bite sustained in Santa Cruz County, Arizona, USA. B) Histological appearance of the eschar biopsy specimen showing ulcerated epidermis with hemorrhage and perivascular lymphohistiocytic inflammatory infiltrates in the superficial dermis. Hematoxylin-eosin staining; original magnification ×50. C) Dense lymphohistiocytic infiltrates around eccrine ducts in the deep dermis of the biopsy specimen. Hematoxylin-eosin staining; original magnification ×100. D) Sparsely distributed intracellular antigens of *Rickettsia parkeri* (red) within the inflammatory infiltrates, detected by immunohistochemistry. Alkaline phosphatase with naphthol-fast red and hematoxylin counterstaining; original magnification ×158.

Patient 1 was a 49-year-old male resident of Arizona. In July 2014, he was hiking in the Pajarito Mountains of Santa Cruz County, Arizona. This remote and semi-arid region receives a mean annual precipitation of 430 mm and is situated at ≈1,200 m above sea level (Figure 1, panel A). During the hike, the man removed and discarded an adult tick he found attached to his right arm. The tick had been attached for <3 hours. A similar tick found crawling on the patient was photographed on the same day (Figure 1, panel B). An ulcerated lesion appeared at the site of the tick bite ≈5 days later. Ten days after the tick bite, the man had onset of fever with a temperature reaching 38.7°C, which was accompanied by headache, myalgia, and scalp tenderness. On day 11, his physician noted a 1-cm eschar, surrounded by a ring of erythema, lateral to the antecubital fossa of his right arm (Figure 2, panel A). No rash or lymphadenopathy was noted. The patient was prescribed doxycycline (100 mg 2×/d for 10 days), and his temperature returned to normal within 24 hours. However, a sparse maculopapular rash subsequently developed on his back, flank, abdomen, and feet; this rash improved within 4 days. The patient reported no recent out-of-state travel or other tick exposures during the several weeks preceding his illness. A medical entomologist (J.W.M.), expert in *Amblyomma* tick identification and familiar with previous specimens in this genus collected from southern Arizona, reviewed the tick photograph associated with the case and, on the basis of the distinctive dorsal ornamentation of the tick and its geographic origin, presumptively determined the specimen to be an adult male tick of the *A.* *triste* species. In July 2015, the patient hiked with several other persons in the Pajarito Mountains, ≈5 miles south of where he had sustained a tick bite the preceding year. He and 1 of his hiking companions (patient 2) were bitten by ticks that visually resembled those observed in 2014. The tick that bit patient 1 in 2015 was attached for <8 hours before it was removed. Patient 1 developed a small, erythematous papule with a central depressed scab at the bite site that healed within several days but remained otherwise asymptomatic.

### Patient 2

Patient 2 was a 42-year-old female resident of Arizona. While hiking, she discovered a tick attached to her scalp behind her right ear. The tick was attached for <8 hours before it was removed. A small ulcer surrounded by a narrow rim of erythema developed at the bite site. Four days after the tick bite, the patient had onset of fever with a temperature of 37.7°C, myalgia, and fatigue. Two days later, a sparse maculopapular rash appeared on her lower legs and arms; this rash lasted for ≈3 days. The patient was prescribed doxycycline (100 mg 2×/d for 10 days) on the first day of fever, and her constitutional symptoms resolved within 48 hours. She did not report any out-of-state or other outdoor exposures during the weeks before her illness.

## Materials and Methods

Two tissue biopsy specimens were collected in July 2014 from the eschar of patient 1. DNA was extracted from 1 sample using a QIAamp DNA Mini Kit (QIAGEN, Valencia, CA, USA) and eluted in a final volume of 200 μL. Extracted DNA was tested in duplicate by using *Rickettsia* genus–specific, *R.* *rickettsii*–specific, and *R. parkeri*–specific real-time PCR assays (*9*,*10*). Cycle threshold (C_t_) values <40 were considered positive. A nested PCR assay was used to amplify a segment of the *ompA* antigen gene by using 3 μL of purified DNA template and 0.8 μmol/L each of primer 190–70 and 190–701 in the primary reaction and 1 μL of the completed primary PCR reaction and 0.8 μmol/L each of primer 190-FN1 and 190-RN1 in the nested reaction (*2*). The amplified DNA fragment was sequenced by using a 3130xl Genetic Analyzer (Applied Biosystems, Foster City, CA, USA). Sequence alignments were made by using SeqMan Pro in the DNASTAR Lasergene 12 suite (DNASTAR, Inc., Madison, WI, USA) and evaluated with BLAST (http://blast.ncbi.nlm.nih.gov/blast.cgi). A second biopsy specimen was fixed in 10% neutral-buffered formalin and embedded in paraffin. Tissue sections cut at 3 μm in thickness were stained with hematoxylin-eosin and tested by using an immunoalkaline phosphatase technique with a polyclonal anti–*R. rickettsii* antiserum, diluted 1:500 (*11*).

Acute- and convalescent-phase serum samples were collected from patient 1 in 2014 and 2015 and from patient 2 in 2015. These specimens were tested for IgG and IgM reactive with antigens of *R. parkeri* and *R.* *rickettsii* by using an indirect immunofluorescence antibody assay. All specimens were diluted initially at 1:32, and antibody titers were expressed as the reciprocal of the last subsequent dilution that provided specific fluorescence. An antibody titer >64 was considered evidence of past exposure to a spotted fever group *Rickettsia* species, and a >4-fold change in titer between specimens collected separately was considered evidence of a recent infection or exposure (*11*). Serum samples were processed by using the Zorba IgG Removal Kit (Zeus Scientific, Branchburg, NJ, USA) before evaluating for IgM.

In 2015, the 2 male ticks that had bitten patients 1 and 2, respectively, and an additional 4 female and male specimens found crawling on clothing of the patients and their hiking companions were placed in 70% ethanol. These specimens were sent to the US Department of Agriculture National Veterinary Services Laboratories (Ames, Iowa, USA) for morphologic identification, and they were subsequently tested at the Centers for Disease Control and Prevention (Atlanta, Georgia, USA) by molecular techniques for evidence of infection with *R. parkeri*. Ticks were minced individually by using sterile scalpel blades. DNA was extracted by using a QIAamp DNA Mini Kit, and each sample was eluted in a final volume of 100 μL. Extracted DNA was evaluated by using a *Rickettsia* genus–specific real-time PCR assay and a nested *ompA* antigen gene PCR assay followed by sequencing, as described previously. An additional collection of 1 male and 1 female *A. triste* tick taken from clothing of the same hikers in Santa Cruz County, Arizona, was retained as a voucher in the parasitology reference collection at the National Veterinary Services Laboratories (accession no. 15-023437).

## Results

Real-time PCR of DNA extracted from the eschar of patient 1 yielded positive results when evaluated using the *Rickettsia* genus–specific assay (averaged C_t_ 36.86, SD 0.74) and the *R. parkeri*–specific assay (C_t_ 35.51) and yielded a negative result when evaluated using the *R.* *rickettsii*–specific assay. A 540-bp segment of the *ompA* gene amplified by a nested PCR assay demonstrated complete identity with the corresponding segment of the *ompA* gene of *R. parkeri* strain Portsmouth (GenBank accession no. CP003341.1). Microscopic examination of the formalin-fixed skin biopsy specimen demonstrated ulceration of the epidermis and lymphohistiocytic inflammatory cell infiltrates distributed predominantly around small blood vessels and eccrine glands and ducts in the superficial and deep dermis. Inflamed vessels revealed focally swollen endothelial cells but no fibrin thrombi (Figure 2, panels B and C). Immunohistochemical staining for spotted fever group *Rickettsia* spp. revealed sparse intracellular antigens of *R. parkeri* within macrophages in the inflammatory infiltrates (Figure 2, panel D).

Serum samples from patient 1, collected 6 and 24 days after the onset of his illness in 2014, reacted with *R. parkeri* antigens at IgG titers of 64 and 512, respectively, and with *R.* *rickettsii* antigens at IgG titers of <32 and 128, respectively. Samples from these 2 collection dates demonstrated IgM titers of 1,024 and 1,024 when these were reacted with *R. parkeri* and *R.* *rickettsii*, antigens, respectively. Serum samples collected from this patient in 2015, at 12 and 34 days after his second tick bite, reacted with *R. parkeri* antigens at IgG titers of 32 and 128, respectively, and with *R.* *rickettsii* antigens at IgG titers of 32 and 128, respectively. No IgM reactive with *R. parkeri* or *R.* *rickettsii* antigens was detected in either sample. Serum specimens collected from patient 2 at 1 and 32 days after the onset of fever reacted with *R. parkeri* antigens at IgG titers of 64 and 64, respectively, and with *R.* *rickettsii* antigens at IgG titers of <32 and <32, respectively. No IgM reactive with *R. parkeri* or *R.* *rickettsii* antigens was detected in either sample.

By using the only published taxonomic key that includes *A. triste* among North American *Amblyomma* spp. ticks (*8*) in addition to 2 widely accepted taxonomic keys to Neotropical ticks (*12*,*13*), each of 2 female and 4 male tick specimens collected in 2015 were identified as *A. triste* on the basis of details of scutal ornamentation, leg armature, and festoons. The *Rickettsia* genus–specific real-time PCR assay was positive for 3 male ticks (average C_t_ 18.36–19.93, SD 0.05–0.09), including the 2 ticks that bit patients 1 and 2. A 540-bp segment of the *ompA* gene was amplified by using nested PCR on each of these same specimens, and sequence analysis demonstrated complete identity with the corresponding segment of the *ompA* gene of *R. parkeri* strain Portsmouth.

## Discussion

Before this report, all documented US cases of *R. parkeri* rickettsiosis occurred within the known geographic range of *A. maculatum* ticks, predominantly in coastal states of the Eastern Seaboard and along the Gulf of Mexico (*1*). The patients described in this report were infected with *R. parkeri* in southern Arizona after bites from ticks identified photographically and morphologically as *A. triste*. To our knowledge, established US populations of the Gulf Coast tick do not occur west of the 100th meridian. In contrast, collection records from multiple sources documented historical attachments of *A. triste* ticks to humans in Cochise (1942) and Santa Cruz (1992) Counties in southern Arizona (*7*). Adult *A. triste* tick collections have been reported from these counties during July–September, corresponding with the local monsoon season.

*A. triste* is an aggressive, human-biting tick species related closely to *A. maculatum* (*14*) and is recognized as a potential vector of *R. parkeri* in Argentina, Brazil, and Uruguay, where rates of rickettsial infection in this tick species range from ≈6% to 20% (*15*–*20*). The distribution of *A. triste* ticks in North America is less well-characterized, with validated collection records from the edges of the Chihuahuan Desert, generally at higher altitudes, and in the Mexican Highlands section of the Basin and Range Province, within the US states of Arizona and Texas (*7*) and the states of Sonora, Durango, and Coahuila in Mexico (*7*,*21*). Our findings indicate that the *A. triste* tick is also a vector of *R. parkeri* in southern Arizona. Although *A. triste* ticks have probably adapted to certain semi-arid environments of the southwest, preliminary observations from this investigation and archival collection records suggest that host-seeking adult *A. triste* ticks are most active during July–September, corresponding to the monsoon season in this region and the period of highest risk for human exposure to *R. parkeri*. *R.* *rickettsii*, the agent of Rocky Mountain spotted fever (RMSF), is also endemic to southern Arizona and northern Mexico, where it is transmitted to humans by *Rhipicephalus sanguineus* ticks (*22*,*23*).

The clinical characteristics of the confirmed and probable cases of *R. parkeri* rickettsiosis described in this report are similar to previous descriptions of the disease (*4*–*6*,*11*). Of particular interest is the re-exposure of patient 1 to an *R. parkeri*–infected tick ≈1 year after primary infection with this agent. During his initial infection in 2014, the patient generated substantial titers of IgG and IgM reactive with antigens of *R. parkeri* and *R.* *rickettsii*. In 2015, after the bite of another infected tick, this patient had a small and rapidly healing lesion at the inoculation site and demonstrated an IgG seroconversion to these same antigens, but did not otherwise become ill and did not mount a measurable IgM response to either antigen. In this context, these data identified an anamnestic antibody response after exposure to an infected tick in 2015 and suggest that some level of protective immunity to *R. parkeri* persisted in patient 1 for at least 1 year after his primary infection.

Future studies should aim to better identify the geographic and host ranges of *A. triste* ticks in the southwestern United States and the frequency with which these ticks are infected with *R. parkeri.* Nonetheless, our data suggest that at least some of the ≈330 cases of RMSF reported from Arizona during the past 10 years (http://www.azdhs.gov/phs/oids/data/stats-archive.htm) might actually represent infections with *R. parkeri*. Because the geographic distribution of *A. triste* ticks also includes several states of northern Mexico, some cases of spotted fever group rickettsiosis in this region might be attributable to infections with *R. parkeri*. Commonly used serologic tests do not distinguish between these clinically similar tickborne diseases, and molecular assays are necessary to provide an etiologic diagnosis (*11*). RMSF is a life-threatening infection that was associated with a 7% case-fatality rate in Arizona during 2002–2011 (*24*) and a 20% case-fatality rate among patients <19 years of age in Sonora, Mexico, during 2004–2013 (*25*). By comparison, no deaths have been attributed to *R. parkeri* rickettsiosis (*1*,*4*,*6*,*11*). Although RMSF and *R. parkeri* rickettsiosis both respond rapidly to therapy with doxycycline, species-specific diagnoses are crucial to accurately define the epidemiologies of the individual diseases in regions where both pathogens might be endemic. 

Identification of *R. parkeri* rickettsiosis in southern Arizona demonstrates a need for local ecologic and epidemiologic assessments to better understand geographic distribution and define public health risk. Education and outreach aimed at persons recreating or working in this region of southern Arizona would improve awareness and promote prevention of tickborne rickettsioses.
